# Digitale Leitlinien des 21. Jahrhunderts: Anforderungen an die strukturierte Entwicklung, Anwendung und Evaluation medizinischen Wissens

**DOI:** 10.1007/s00101-023-01310-3

**Published:** 2023-06-16

**Authors:** Patrick Meybohm, Alexander Ghanem, Falk von Dincklage, Peter Kranke, Philipp Börm

**Affiliations:** 1 Klinik und Poliklinik für Anästhesiologie, Intensivmedizin, Notfallmedizin und Schmerztherapie, Universitätsklinikum Würzburg, Oberdürrbacher Str. 6, 97080 Würzburg, Deutschland; 2 Innere Medizin II, Asklepios Klinik Nord-Heidberg, Hamburg, Deutschland; 3 Klinik für Anästhesie, Intensiv‑, Notfall- und Schmerzmedizin, Universitätsmedizin Greifswald, Greifswald, Deutschland; 4 Börm Bruckmeier Verlag GmbH, Grünwald, Deutschland

**Keywords:** Leitlinien, Entscheidungsunterstützungssystem, Digitalisierung, Interoperabilität, Medizinprodukt, Guidelines, Decision support system, Digitalization, Interoperability, Medical device

## Abstract

Bislang werden klinische Leitlinien als verallgemeinerte Darstellungen klinischen Wissens verstanden, die nach bester verfügbarer Evidenz die Anforderungen an die Versorgung von Patienten in spezifischen Patientensituationen aufzeigen. In diesem Expertenmeinungsartikel soll erörtert werden, wie digitale Leitlinien beschaffen sein müssten, und welche Anforderungen an die strukturierte Entwicklung, Anwendung und Evaluation solcher Leitlinien gestellt werden müssten. Eine Digitalisierung von Leitlinien muss die Transformation analoger, textbasierter Leitlinieninformationen in Formate berücksichtigen, die über Benutzeroberflächen (Interfaces) eine Mensch-Maschine-Interaktion ermöglichen, Ärzten die Anforderungen an eine leitlinienkonforme Patientenversorgung aufzeigen und die außerdem Maschinenspeicherung, Maschinenausführung und Maschinenverarbeitung von Patientendaten ermöglichen.

## Abstract (en)

Until now, clinical guidelines have been understood as generalized representations of clinical knowledge that, based on best available evidence, show the requirements for patient care in specific patient situations. This expert opinion article is intended to discuss how digital guidelines should be designed and which requirements must be met for the structured development, application and evaluation of such guidelines. The digitalization of guidelines must take into account the transformation of analogue text-based guideline information into formats that enable human-machine interaction via user interfaces, that show physicians the requirements for guideline-compliant patient care and that also enable machine storage, machine execution and machine processing of patient data.

## Einführung

2008 schilderte Peter Waegemann, damals Direktor des Medical Records Institute in Boston, in einem Interview seine Vision der Medizin von morgen: *„We live in an age where people don’t use maps any more. They use GPS systems which tell them turn left, turn right. And this is how health care is going to work. […] The smart boy of the future will not be the person who can memorize everything, but who can navigate the system“* [1].

In einem solchen *Navigationssystem für Ärzte* würden das Wissen und die Informationen, die für die Versorgung der Patienten in einer konkreten Situation relevant sind, stets *unabhängig von Arzt* und Vorwissen des Arztes am Versorgungsort zur Verfügung stehen. Ein solches System wäre nicht mehr text-, sondern *softwarebasiert* und würde den Arzt Schritt für Schritt durch den Versorgungsprozess des Patienten navigieren. Der Arzt würde seine Entscheidungen dann stets so treffen und Patienten so versorgen, wie diese es erwarten dürfen: nämlich auf die Art und Weise, die bei Patienten in ähnlichen Situationen in der Vergangenheit die besten Ergebnisse gezeigt haben, für die also die beste verfügbare Evidenz vorliegt. Die Rolle des Arztes bestünde dann insbesondere im Einordnen des Wissens und seiner kontextabhängigen Interpretation unter Berücksichtigung der Lebenssituation des Patienten und seiner individuellen Wertevorstellungen. Peter Waegemanns Vision eines Navigationssystems für Ärzte aus dem Jahr 2008 blieb bisher aber leider unerfüllt. Sie blieb bislang eine Utopie!

Im Folgenden soll nun erörtert werden, welche *Voraussetzungen* aus Sicht der Autoren erfüllt sein müssten, um dieser digitalen Utopie näherzukommen. Wie können klinisches Wissen und insbesondere klinische Leitlinien für so ein System digital erfasst und abgebildet werden? Welche Infrastruktur müsste vorhanden sein, um klinisches Wissen darin nicht mehr nur rein textbasiert, sondern wirklich digital zu entwickeln, am Versorgungsort digital anzuwenden und die Ergebnisse (Patienten-Outcomes) schließlich auch digital zu evaluieren? Wie könnten die Prozesse der Entwicklung, Anwendung und Evaluation strukturiert dargestellt werden? Und wie könnten Abweichungen von idealtypischen Versorgungsszenarien – zum Beispiel aufgrund der Einbeziehung der Patientenperspektive – dokumentiert und evaluiert werden?

Bislang werden *klinische Leitlinien* als verallgemeinerte Darstellungen klinischen Wissens in Form von Texten verstanden, die nach bester verfügbarer Evidenz die Anforderungen an die Versorgung von Patienten in spezifischen Patientensituationen aufzeigen, d. h. die Versorgungsoptionen, deren Risiken und Nutzen sowie effiziente Handlungsabläufe. Eine *Digitalisierung von Leitlinien* muss die Transformation analoger, textbasierter Leitlinieninformationen in Formate berücksichtigen, die über Benutzeroberflächen (Interfaces) für eine Mensch-Maschine-Interaktion geeignet sind, die Ärzten die Anforderungen an eine leitlinienkonforme Patientenversorgung aufzeigen und die gleichzeitig die Möglichkeiten einer Maschinenspeicherung, Maschinenausführung und Maschinenverarbeitung der Patientendaten aufweisen.

Wie kommen wir also von textbasierten S1-, S2- und S3-Leitlinien, von sog. Clinical Practice Guidelines, zu benutzerfreundlichen digitalen Systemen? Und zwar ohne die Wertigkeit und Vertrauenswürdigkeit der Leitlinienaussagen dabei einzubüßen. Dies sei die zentrale Frage, die im Folgenden aus Sicht der Autoren erörtert werden soll.

## Anforderungen an die Digitalisierung klinischer Leitlinien

### Benutzerfreundlichkeit, Praktikabilität (Usability)

Leitlinien sollten zuallererst prägnant, offensichtlich hilfreich und praxisnah sein. Einfach zu bedienende Benutzeroberflächen sollten einen schnellen Zugriff auf das Handlungswissen am Versorgungsort erlauben und schnelle, sofort anwendbare Anleitungen geben. Leitlinien sollten aus der Sicht des anwendenden Arztes entwickelt und gedacht werden. Der Gesetzgeber fordert beispielsweise, dass Medizinprodukte auf Gebrauchstauglichkeit (engl. „usability“) getestet werden! [2] Warum nicht auch Leitlinien?

### Operationalisierung, Interaktivität

Digitale Leitlinien sollten interaktiv über Mensch-Maschine-Interfaces funktionieren. Sie sollten bei der Benutzung nur die Patientendaten abfragen und nur das Wissen demonstrieren, das im jeweils konkreten Patientenfall auch relevant ist. Die nichtrelevanten Anteile der Leitlinie, die im konkreten Kontext nicht benötigt werden, sollten auch nicht angezeigt werden. Das wären dann maßgeschneiderte Präzisionsleitlinien. Die Anwendung der Leitlinien würde dadurch auch sicherer, da der Arzt nichtpassende Leitlinientexte gar nicht erst lesen bzw. irrelevante Patientendaten nicht zu erfassen bräuchte. Der Arzt könnte sich auf Wesentliches konzentrieren und hätte mehr Zeit für die eigentliche Patientenversorgung. Es werden also operationalisierte Leitlinien benötigt.

### Prozessorientiertheit

So, wie Qualität und Qualitätsmanagement (ISO 9001:2015) prozessorientiert sind, sollten auch Leitlinien einen klaren Ablauf der erforderlichen Aktivitäten aufzeigen. Leitlinien sollten den Arzt in der Patientenversorgung dadurch schnell zu genau dem Punkt führen, bei dem alle Leitlinienanforderungen im spezifischen Patientenfall erfüllt sind. Alle für die Versorgungsentscheidung erforderlichen Patientencharakteristika wären dann geklärt, und es könnten genau solche Versorgungsoptionen ausgewählt werden, die die Leitlinie als indiziert empfiehlt. Prozesse können generell in Form von Entscheidungsbäumen und Tabellen aufgezeigt werden. In digitalen Leitlinienartefakten könnten die einzelnen Prozessschritte auf der Benutzeroberfläche und in der richtigen Prozessreihenfolge visualisiert, d. h. klickbar dargestellt werden. Dadurch könnte der Prozess der erforderlichen Aktivitäten dem versorgenden Arzt erfahrbar gemacht werden.

### Risikoorientiertheit, Nutzen-Risiko-Analyse

Leitlinien sollten Nutzen und Risiken der einzelnen Interventionen aufzeigen, d. h. klare Nutzen-Risiko-Analysen für die jeweiligen Versorgungsoptionen liefern [3]. Leitlinien könnten dann auch als Risikovorhersageinstrumente, also als Prädiktionsinstrumente, angesehen werden, die Ärzte unterstützen, evidenzbasiert Diagnostik- und Behandlungsoptionen anzuwenden. Nutzen und Risiken der Interventionen könnten dann stets und auf der Basis der besten zur Verfügung stehenden Evidenz mit Patienten besprochen werden, sodass ein echtes „Shared Decision-Making“ möglich wird.

### Kosteneffizienz, Parsimonität

Leitlinien sollten ohnehin von Anfang an die Kosten und die Verfügbarkeit der benötigten medizinischen Ressourcen berücksichtigen [4]. Leitlinien könnten die schrittweise Erfüllung der Anforderungen aber auch so vorausdenken und planen, d. h. klinische Prozesse so modellieren, dass sie dazu beitragen, unnötige und potenziell teure klinische Maßnahmen zu vermeiden, z. B. kostspielige, nichtindizierte Untersuchungen. Auf diese Weise würde das Evidence to Decision Framework in die Navigation zur Leitlinienumsetzung integriert [5]. Hilfreich ist ferner auch die Option, eine „zweitbeste“ Diagnostik- oder Therapieoption visualisiert zu bekommen. Dann nämlich, wenn diagnostische Optionen nicht zur Verfügung stehen, Kontraindikationen bestehen oder vom Patienten auch dezidiert abgelehnt werden.

### Leistungskennzahlen, Auditierbarkeit

Außerdem sollte bereits in den digitalen Leitlinien beschrieben werden, wie die Leistungserbringung klinischer Verfahren durch definierte Leistungsindikatoren (engl. „key performance indicators“) im Nachhinein gemessen werden können [6, 7]. Diese Messgrößen könnten Indikatoren zu Qualität, Effizienz, Sicherheit, Transparenz und Outcomes (QESTO-Indikatoren) umfassen [8]. Lägen Leitlinien in Form von Software-Interfaces digitalisiert vor und würden Leitlinien digital, d. h. durch Anklicken dieser Oberflächen, angewendet, dann wäre die Leistungserbringung augenblicklich messbar, quantifizierbar und somit auch auditierbar. Ein digitales Leitliniensystem könnte eine regelmäßige Überprüfbarkeit (Auditierbarkeit) der klinischen Leistungen [9], d. h. die Erfüllung vorgegebener Anforderungen (z. B. Leitlinienadhärenz), überhaupt erst ermöglichen.

### Transparenz

Jede in eine konkrete Handlung übersetzbare Leitlinienaussage sollte durch ein Aussagenprofil ergänzt werden, in dem in transparenter Weise und per Klick leicht zugänglich Zusatzinformationen zu Nutzen, Risiken, zu deren Häufigkeiten/Wahrscheinlichkeiten, zu Evidenz- und Empfehlungsgraden, aber auch zu Literaturquellen etc. aufgelistet sind. In digitalen Leitlinieninstrumenten kann dann sogar dargestellt werden, dass im spezifischen Patientenfall für keine Intervention Evidenz vorhanden ist und somit auch kein Procedere vorgeschlagen werden kann. Durch farbliche Differenzierungen, Piktogramme etc. könnten schon auf dem User-Interface unterschiedliche Evidenz- und Empfehlungsgrade, d. h. auch „Kann-Empfehlungen“, visualisiert werden. Der Arzt muss verstehen können, warum eine spezifische Versorgungsoption im konkreten Patientenfall indiziert ist bzw. vorgeschlagen wird. Nachversorgende Ärzte sollten ebenfalls die Gründe für Auswahl oder Nichtauswahl der durchgeführten Intervention – nicht zuletzt aufgrund von Patientenpräferenzen – erkennen können. Diese Transparenz könnte durch ein digitales Leitliniensystem gewährleistet werden.

### Datenmodell, Interoperabilität, Implementierbarkeit, Erforschbarkeit

Leitlinien sollten bereits mit ihrer Veröffentlichung ein problemspezifisches *Datenmodell* abbilden, das eine automatisierte Weiterverarbeitung erlaubt. Nur so lässt sich der Prozess der Bereitstellung des Wissens aus den Leitlinien von dem Prozess der Einbindung des Wissens in Softwaresysteme trennen [10]. Durch eine solche Trennung ließe sich verhindern, dass eine Leitlinie für jedes Softwaresystem erneut in ein spezifisches maschineninterpretierbares Format übersetzt werden muss und dabei jedes Mal das Risiko von Übertragungsfehlern entsteht. Leitlinien sollten also schon von Anfang an in digitaler, vollständig interoperabler, maschinenlesbarer und maschinenausführbarer Form erstellt werden, um die Anwendbarkeit in computergestützten klinischen Umgebungen zu ermöglichen [11]. Zur Abbildung der Logik von Leitlinienempfehlungen bedarf ein Datenmodell zumindest der Beschreibung der Patientencharakteristika (PICO-P), auf die eine Empfehlung angewendet werden soll, sowie der empfohlenen Interventionen (PICO-I). Zur Gewährleistung der *Interoperabilität* sollte die Modellierung gemäß aktueller Standards erfolgen (z. B. HL7 FHIR, EBM-on-FHIR, CPG-on-FHIR) [12]. Doch damit nicht nur die Logik, sondern auch die begrifflichen Inhalte einer Empfehlung automatisiert durch einen Computer verstanden werden können, bedarf es darüber hinaus auch einer Verwendung eindeutig definierter Begriffe unter Nutzung international standardisierter Formate (z. B. SNOMED CT, LOINC, ICD 10, CTCAE). Die Anwendung vollständig computerverständlicher digitaler Leitlinienderivate bietet dann auch die Möglichkeit der Generierung von Realweltdaten und Realweltevidenz, um den Lebenszyklus klinischen Wissens zu beschleunigen.

### Interoperabilität der Leitlinieninhalte

Während der Anwendungsbereich aktueller Leitlinien meistens auf ein einziges Patientenproblem, z. B. eine Erkrankung oder eine Diagnose, beschränkt ist, sollten künftige Leitlinien noch mehr die relevanten Aspekte der Multimorbidität (Komorbiditäten) und Polypharmazie von Patienten berücksichtigen. Leitlinien sollten interoperabel zu anderen Leitlinien sein, d. h. zu den verallgemeinerten Darstellungen der Versorgungsanforderungen bei anderen Patientenproblemen. Leitlinien sollten sich also digital ergänzen können.

### Vigilanzsystem („Post-Market Surveillance of Guidelines“), Warnhinweissystem

Digitale Leitlinien sollten eine kontinuierliche Feldüberwachung und eine systematische Identifizierung von Sicherheitsproblemen bei der Anwendung der Leitlinienempfehlungen ermöglichen. Ein digitales Leitliniensystem sollte kontinuierlich Feedback der Anwender einsammeln, strukturiert abspeichern und zu gegebener Zeit (oder auch sofort) an die Leitlinienentwickler weiterleiten können. Leitlinienautoren könnten das Feedback dann auswerten und bei der Entwicklung nachfolgender Versionen der Leitlinien berücksichtigen; ganz im Sinne eines kontinuierlichen Verbesserungsprozesses. Bei besonders relevanten Sicherheitsproblemen könnten aber auch Warnhinweise an die Nutzer der digitalen Leitlinien, d. h. an die Ärzte, zurück übermittelt werden. Ein digitales Leitliniensystem für Ärzte könnte als Leitlinien-Vigilanz-System und auch als Warnhinweis-Kommunikation-System genutzt werden. Die Beachtung eines „Rote-Hand-Briefes“ oder einer „Black Box Warning“ würde nicht mehr dem Zufall überlassen, sondern integraler Bestandteil eines Entscheidung-Unterstützung-Systems werden.

### Fortlaufende Evaluation

Leitlinien sollten durch Leitlinienentwickler regelmäßig, eigentlich fortlaufend, neu evaluiert und weiterentwickelt werden. Dabei sollten neue Forschungsergebnisse, neue Evidenzen, aber auch Rückmeldungen (Feedbacks) der nutzenden Ärzte berücksichtigt werden. Um eine kontinuierliche Verbesserung der Leitlinien zu gewährleisten, könnten wie bei den Impfempfehlungen der STIKO „ständige Kommissionen“ eingerichtet werden, die die Leitlinien laufend verbessern (Engl: „living guidelines“). Durch ein digitales Leitliniensystem könnten die neu erarbeiteten Leitlinien oder Leitlinienaussagen auch schnell wieder an anwendende Ärzte weitervermittelt werden.

### Lebenszyklus klinischen Wissens (dynamisches Leitlinienmodell)

**Figure 1:**
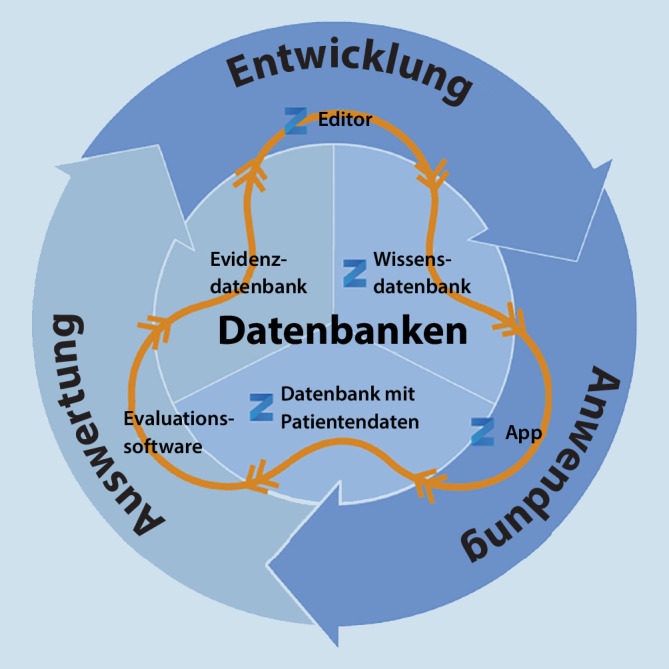

Die digitalen Formate klinischen Wissens müssen passend und nutzbar sein, in allen Lebensstufen klinischer Leitlinien und klinischer Leitlinienaussagen. Der Lebenszyklus klinischen Wissens umfasst die drei Phasen Entwicklung des Wissens, klinische Anwendung und Auswertung (Evaluation) der Ergebnisse. Die digitalen Formate der Wissensartefakte sollten in allen Phasen des Lebenszyklus klinischen Wissens funktionieren. Alle drei Phasen müssen digital abgebildet werden können (Abb. 1).

In der *ersten Phase des Lebenszyklus klinischen Wissens*, der *Entwicklung*, entsteht neues klinisches Wissen. Einzelne (Leitlinien‑)Therapie-Aussagen werden erarbeitet und in einer Wissensdatenbank (Leitliniendatenbank) abgespeichert.

In der *zweiten Phase*, der* Anwendung*, wird das Wissen dann im klinischen Alltag angewendet. Leitlinientexte werden von Ärzten gelesen und verarbeitet. Ein klinisches Navigationssystem für Ärzte könnte interaktiv Therapieempfehlungen anzeigen. Die ausgewählte Versorgungsoption wird dann am jeweiligen Versorgungsort am Patienten angewendet. Während dieser Anwendung des klinischen Wissens entstehen Anwendungsdaten. Diese Patientendaten (Realweltdaten) können in einer Patientendatendatenbank abgespeichert werden.

In der *dritten Phase *des Lebenszyklus klinischen Wissens, der *Evaluation*, werden die gesammelten Patientendaten dann ausgewertet, Zusammenhänge werden gesucht. Für bestimmte Patientensituationen wird dann – aufgrund vorliegender Outcome-Daten – die Art und Weise der Versorgung erkannt, die auch in der Zukunft bei Patienten in ähnlichen Situationen die besten Ergebnisse zeigen wird, für die also eine verbesserte verfügbare Evidenz vorliegt. Die neu generierte Evidenz (Real-Welt-Evidenz) kann in einer Evidenzdatenbank gesammelt werden.

Jedem Lebenszyklus klinischen Wissens schließt sich gleich der nächste Zyklus an, mit den gleichen Phasen, nämlich der Weiterentwicklung des Wissens, der erneuten Anwendung und dann wiederum einer Evaluation. Je schneller diese Zyklen ablaufen, desto schneller können neue Erkenntnisse entstehen, desto schneller können Ergebnisse auch gleich wieder in der Patientenversorgung angewendet werden. Dieses *dynamische Leitlinienmodell*, das den Lebenszyklus klinischen Wissens berücksichtigt, sollte ebenfalls bei der Digitalisierung klinischer Leitlinien berücksichtigt werden.

### Erstattung, Gesetzgebung

Digitale Leitlinien könnten es außerdem der Gesetzgebung ermöglichen, Erstattungssysteme mit Anreizen für die Bezahlung einer evidenzbasierten Praxis einzuführen. So könnte die Benutzung digitaler Leitlinien durch Zusatzentgelte vergütet werden. Dies könnte z. B. auch nach dem Gesundheitsversorgungsweiterentwicklungsgesetz [13] durch Qualitätsverträge in den vom G‑BA noch festzulegenden Leistungsbereichen erprobt werden.

### Regelbasierte Entscheidungsunterstützungssysteme, EU-Regulatorik, bestehende PDMS-Systeme

Die in diesem Artikel beschriebenen digitalisierten Leitlinien der Zukunft sind, im Grunde genommen, eine Kernkomponente für eine leitlinienbasierte klinische Entscheidungsunterstützung (engl. Clinical Decision Support, CDS). Einzelne textbasierte Leitlinienaussagen werden dabei in digitale Entscheidungsregeln übersetzt und dann in größere, z. T. komplette Leitlinien umfassende CDS-Tools integriert. Bei Systemen, die derartige digitale Leitlinien der Zukunft einbinden, handelt es sich also um regelbasierte und nicht um AI-basierte Entscheidungssysteme. Jede Empfehlung, d. h. Ausgabe des Systems, kann dann durch das System auch ganz klar durch passende Leitlinienaussagen begründet werden.

CDS-Tools und CDS-Systeme unterliegen in der EU der Medizinprodukteverordnung (MDR) und benötigen eine spezifische CE-Zertifizierung. Im Wesentlichen muss der technische Entwickler des die digitalen Leitlinien nutzenden Systems, d. h. der Inverkehrbringer des CDS-Medizinprodukts, ein Qualitätsmanagementsystem nach ISO 13485 betreiben und eine MDR-konforme technische Dokumentation des Systems anfertigen. Zur MDR-konformen technischen Dokumentation gehören u. a. eine klinische Evaluation, d. h. der Nachweis des klinischen Nutzens und der Sicherheit des Produkts, sowie eine Validierung der Benutzerfreundlichkeit des Systems. Die sog. CE-Zertifizierung (Medizinprodukt) umfasst außerdem die jährliche Auditierung des Medizinproduktherstellers durch eine benannte Stelle, z. B. durch die TÜV Süd GmbH. Möglicherweise sollten also für die Entwicklung von auf digitalen Leitlinien basierenden Entscheidungsunterstützungssystemen CE-zertifizierte Spezialanbieter hinzugezogen werden, die in der Lage sind, die regulatorischen EU-Anforderungen zu erfüllen.

Solange solche CE-Zertifizierungen jedenfalls nicht für bestehende PDMS-Systeme und die darin anzuwendenden CDS-Tools vorliegen, scheinen die hier beschriebenen digitalen Leitlinien auch nicht in diesen angewendet werden zu können. Als weitere Hürde für die Implementierung von auf digitalen Leitlinien basierender CDS-Software in bestehende PDMS-Systeme müssten die dort enthaltenen Patientendaten in hochgranulärer, semantisch kodierter und strukturell interoperabler Form vorliegen. Dies ist jedoch in aktuell eingesetzten Patienten-Daten-Management-System (PDMS) in der Regel nicht der Fall. In diesen Systemen, deren primäre Entwicklung teilweise auf Kernkomponenten aus den 80er- und 90er-Jahren des 20. Jahrhunderts basieren, liegen die Patientendaten oft nur in proprietären und nichtstandardisierten Formaten vor. Es müssten also wohl neue, CE-zertifizierte PDMS für die Anwendung digitaler Leitlinien entwickelt werden, in denen Interoperabilität und regulatorische Anforderungen berücksichtigt werden. Dies umfasst natürlich auch den Datenschutz.

## Fazit

Für den Erfolg der Digitalisierung klinischen Wissens, insbesondere für den Erfolg der digitalen Evaluation, Weiterentwicklung und Anwendung klinischer Leitlinien, ist es langfristig wohl unerlässlich, die genannten Anforderungen und Voraussetzungen für echte digitale Leitlinien des 21. Jahrhunderts zu erfüllen. Bei der Übersetzung von Leitlinientexten in digitale Instrumente werden wohl Methoden der künstlichen Intelligenz, des Machine Learning und des Natural Language Processing zum Einsatz kommen und die Prozesse vereinfachen. Die Einzelaufgaben der Digitalisierung müssen aber trotzdem abgearbeitet werden, auch wenn dies einen hohen Aufwand erfordert [14]. Die Vision des von Peter Waegemann vor mehr als 15 Jahren skizzierten Navigationssystems für Ärzte, *„who can navigate the system“*, wird wohl erst dann realisierbar sein, wenn zumindest ein Großteil der genannten Voraussetzungen erfüllt ist.
